# Genetic variation in the cellular response of *Daphnia magna* (Crustacea: Cladocera) to its bacterial parasite

**DOI:** 10.1098/rspb.2010.0772

**Published:** 2010-06-02

**Authors:** Stuart K. J. R. Auld, Jennifer A. Scholefield, Tom J. Little

**Affiliations:** School of Biological Sciences, Institute of Evolutionary Biology, University of Edinburgh, Ashworth Labs, West Mains Road, Edinburgh EH9 3JT, UK

**Keywords:** invertebrate immunity, haemocytes, host–parasite coevolution, resistance, *Daphnia, Pasteuria*

## Abstract

Linking measures of immune function with infection, and ultimately, host and parasite fitness is a major goal in the field of ecological immunology. In this study, we tested for the presence and timing of a cellular immune response in the crustacean *Daphnia magna* following exposure to its sterilizing endoparasite *Pasteuria ramosa*. We found that *D. magna* possesses two cell types circulating in the haemolymph: a spherical one, which we call a granulocyte and an irregular-shaped amoeboid cell first described by Metchnikoff over 125 years ago. *Daphnia magna* mounts a strong cellular response (of the amoeboid cells) just a few hours after parasite exposure. We further tested for, and found, considerable genetic variation for the magnitude of this cellular response. These data fostered a heuristic model of resistance in this naturally coevolving host–parasite interaction. Specifically, the strongest cellular responses were found in the most susceptible hosts, indicating resistance is not always borne from a response that destroys invading parasites, but rather stems from mechanisms that prevent their initial entry. Thus, *D. magna* may have a two-stage defence—a genetically determined barrier to parasite establishment and a cellular response once establishment has begun.

## 1. Introduction

Parasites often impose substantial costs on their hosts, as evidenced both by the severe effects they can have on individuals, and in the impact they may have on host population sizes (Van Alfen *et al*. 1975; Hudson *et al*. 1998; Duncan & Little 2007). Host defence mechanisms, therefore make a key contribution to organismal fitness and genetic variation for these mechanisms may contribute to host evolution in the face of parasitism. The first line of defence for the invertebrate host often consists of the barrier defences of the cuticle or more complex defences of the gut epithelium (Artis 2008). After these come the haemolymph-based immune defences, for example, phagocytic haemocytes, antimicrobial peptides or lysozymes (Hoffmann 2003; Mydlarz *et al*. 2006). Much of our understanding of invertebrate immunity is built on studies of insect–parasite systems, although there are notable exceptions (Mydlarz *et al*. 2006). We argue the importance of strengthening our knowledge of invertebrate immunity beyond the insects, as well as the need to develop deep understanding of the interplay between naturally coevolving antagonists.

One of the goals of ecological immunology is to determine the role immunological mechanisms play in mediating variation in fitness when organisms are exposed to parasites. To address the function that immune responses have in determining infection outcomes and, ultimately, the fitness consequences of infection (or self-harm owing to immunopathology), it is necessary to measure how immune effector systems vary under genetic and environmental variation. However, many studies aiming to elucidate immune mechanisms have done so in the absence of pathogens, under controlled laboratory conditions and in homogeneous, inbred genetic backgrounds. Thus, while providing the necessary mechanistic backbone for studying the immune function, this approach does not address variation in natural populations (Little *et al*. 2005). However, a considerable body of evidence suggests that the impact of genetic and environmental variation on infection is substantial (Mydlarz *et al*. 2006; Lazzaro & Little 2009), and it is thus difficult to extrapolate from laboratory measures of immune responsiveness to variation in fitness (Viney *et al*. 2005).

Here, we tested for a cellular immune response in a naturally coevolving host–parasite model: the aquatic crustacean, *Daphnia magna* and its sterilizing bacterial endoparasite, *Pasteuria ramosa*. The fitness consequences, for example, host sterilization or mortality due to *P. ramosa* infection have been extensively studied under genetic and environmental variation (Mitchell *et al*. 2005; Duncan *et al*. 2006; Vale *et al*. 2008; Vale & Little 2009), but the mechanisms of resistance have received less attention in this system (Mucklow & Ebert 2003; Mucklow *et al*. 2004; Labbe *et al*. 2009). Circulating haemocytes are an important anti-parasite defence in many invertebrates (Ataev & Coustau 1999; Elrod-Erickson *et al*. 2000; Kraaijeveld *et al*. 2001; Canesi *et al*. 2002; Cotter *et al*. 2004), and have been found in *D. magna* (Metchnikoff 1884). They are central to the innate immune system, being involved in phagocytosis and encapsulation; they are also vehicles for other immune functions, e.g. the generation of reactive oxygen and nitrogen species, as well as antimicrobial peptides and phenoloxidase (Strand 2008). For these reasons, we chose them as the immune marker for this study. Both the induction of a cellular response and its magnitude are likely to contribute to host fitness when the host is in the presence of parasites.

This study also examines how the magnitude of cellular response varies across multiple host genotypes. By embracing host genetic variation, we hope to gain further insight into how parasitism could influence host genetic structure, and ultimately, host evolution. We also test how infection outcome differs across host genotypes, allowing us to link our measures of cellular response with susceptibility. Finally, we sought to determine whether it is the mere presence of parasite spores in the gut, or the process of spores moving from the gut to haemolymph that elicits a cellular response in the host.

## 2. Material and methods

### (a) Host and parasite organisms

*Daphnia magna* is a freshwater crustacean of shallow, eutrophic ponds. It reproduces by cyclical parthenogenesis, where apomictic parthenogenesis is the main reproductive mechanism, but bouts of sexual reproduction occur in the presence of specific cues (Carvalho & Hughes 1983; Hobaek & Larsson 1990; Slarsarczyk *et al*. 2005). By keeping *D. magna* in the absence of sexual cues, purely clonal lines can be maintained in the laboratory.

*Pasteuria ramosa* is a spore-forming, bacterial endoparasite, obligate to *D. magna*. It is transmitted horizontally from dead, infected hosts (Ebert *et al*. 1996), and is believed to infect via the gut and proliferate in the host's haemolymph. Successful *P. ramosa* infections have a profound impact on host fitness, often causing complete host sterilization and premature death (Ebert *et al*. 1996).

Twelve of the 16 host genotypes used here were founded from a single animal, hatched from an ephippium (sexually produced resting egg) in the laboratory. Ephippia were from pond mud collected in Gaazerfeld, Germany in 1997. The other four genotypes (numbers 3, 4, 7 and 13) were also founded from single individuals, but these were collected as adults from Gaazerfeld in 1997 and have since been kept in a state of clonal reproduction. The *P. ramosa* isolate originated from a single infected *D. magna* from that same pond (Carius *et al*. 2001), and has been used in a variety of experiments since that time. The *P. ramosa* spore solution used here was made by homogenizing previously infected hosts with ddH_2_O.

### (b) Experimental set-up

Independent replicates for each *D. magna* genotype were maintained for three generations to minimize variation in condition. Animals were kept in jars containing 200 ml of artificial medium (Kluttgen *et al*. 1994) modified using one-twentieth of the recommended SeO_2_ concentration (Ebert *et al*. 1998) and fed 5.0 ABS *Chlorella vulgaris* algal cells per day (ABS is the optical absorbance of 650 nm white light by the *Chlorella* culture). Their medium was refreshed three times per week. There were five *Daphnia* per jar and jars were incubated at 20°C on a 12L∶12D light cycle. The second-clutch neonates from the third generation were used in each of the four experiments.

The first experiment examined host cellular response in four host clones or genotypes. For this four-genotype cell experiment, replicates were allocated to one of two parasite treatments: non-exposed or parasite-exposed. Parasite treatment lasted for 2 h, 4 h, 6 h or 8 h. Thus, there were six replicates per genotype, per parasite treatment, per time treatment. The second and third experiments both studied 16 genotypes: the second experiment examined host cellular response and the third experiment measured infection outcome. Like the previous four-genotype cell experiment, replicates were allocated to one of two parasite treatments (non-exposed or parasite-exposed), however all replicates were exposed for the same amount of time: 5 h. There were six and twelve replicates per parasite treatment, per genotype for the second and third experiment, respectively. Finally, a fourth experiment used one genotype (genotype 4 from the previous experiment) to test for the presence of a cellular response when the host was exposed to killed (non-infective) spores or live (infective) spores. Spores were killed by heating them in a water bath at 95°C for 30 min. Replicates were allocated to three treatments: non-exposed and parasite-exposed, and exposed to killed parasites. There were eight replicates per treatment.

Parasite exposures were carried out as follows. When at least three out of five of the *Daphnia* in a replicate had deposited eggs in their brood chamber, the replicate was exposed to its parasite treatment. The five *Daphnia* of the replicate were placed together in a well of a 24-well cell plate (Costar, Corning Inc., NY, USA). Parasite-exposed replicates received 50 000 *P. ramosa* spores from the pre-prepared solution. Non-exposed control replicates received the same concentration of uninfected *D. magna* homogenized in ddH_2_O.

### (c) Haemocyte collection and counting

**Figure 1.**
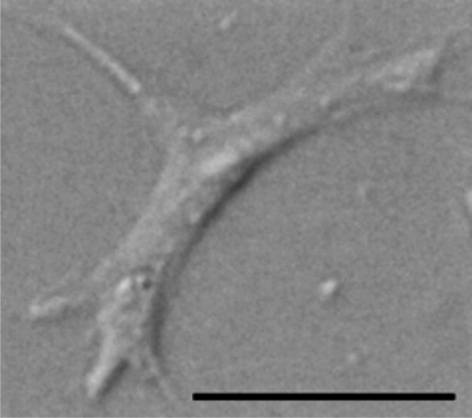
Differential interference contrast image of an amoeboid haemocyte from *D. magna*. Scale bar, 5 µm.

After parasite treatment, five *Daphnia* from each replicate were placed in a cell extraction chamber containing 4.0 µl of ice-cold anticoagulant buffer (98 mM NaOH, 186 mM NaCl, 17 mM EDTA and 41 mM citric acid, pH adjusted to 4.5: Lavine *et al*. 2005). A 25-guage needle (BD Microlance, Drogheda, Ireland) was used to pierce the *Daphnia* heart, causing haemolymph to pool into the medium. The *Daphnia* were then removed and the haemolymph solution was mixed thoroughly using a pipette. Four microlitres of the cell suspension were placed in a fertility counting chamber (0.001 mm^2^ × 0.100 mm (depth); Hawksley, Lancing, Sussex, UK), and the number of amoeboid haemocytes was counted (figure 1). The number of granulocytes did not vary between treatments in any of the cell experiments and are not discussed further. Haemocyte counts were converted to number of cells per microlitre of haemolymph–buffer solution.

### (d) Life-history assays

After parasite treatment, one of the five *Daphnia* from each replicate of the 16-clone life-history experiment was randomly selected and kept individually in 60 ml of artificial medium and fed 1.0 ABS *C. vulgaris* cells per day. Their medium was refreshed three times per week, or after the *Daphnia* had a clutch of offspring, and jars were incubated at 20°C on a 12L∶12D light cycle. Jars were checked daily for clutches and the number of offspring was recorded at each clutch. From day 25 post-parasite exposure, hosts were examined for symptoms of *P. ramosa* infection. Symptoms include cessation of reproduction, absence of ovaries and bacterial growth in the haemolymph. The experiment ran for 32 days.

### (e) Statistical analyses

Data were analysed using R (Ihaka & Gentleman 1996; R Development Core Team 2005). To achieve normality of distribution in the data, haemocyte counts were log-transformed for the four genotype and 16-genotype cell experiments and square-root transformed for the killed parasite cell experiment. For the four-genotype cell experiment, we tested the fixed effects of host genotype, parasite treatment and exposure time, as well as all interaction terms. For the 16-genotype cell experiment, we tested the fixed effects of host genotype and parasite exposure along with their interaction. Welch's two sample *t*-tests were performed *post hoc* on the 16-genotype cell data to test for the presence of a significant cellular response in each of the host genotypes, and the results were corrected for multiple comparisons (Holm 1979). For the killed parasite experiment, we tested for differences between parasite–exposure treatments.

We report the full statistical models for both the four-genotype and 16-genotype cell data, along with the proportion of the variance explained by each of the terms in the full model. Variance proportions were calculated by dividing the sequential sum of squares for each term by the total sum of squares for the model. We then multiplied these proportions by 100 to find the percentage variance explained by each term.

## 3. Results

### (a) Four-genotype cell experiment

**Figure 2.**
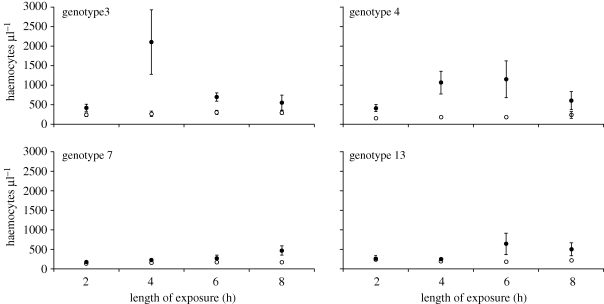
Haemocyte counts per host in *P. ramosa-*exposed and control *D. magna* (filled and open symbols, respectively; *n* = 6 and each replicate consists of five *Daphnia*). Error bars are 1 s.e.m. See table 1 for statistical details.

Haemocyte counts were obtained from 240 *Daphnia* from 48 jars. Averaging across all genotypes, mean circulating haemocyte number per microlitre from the *P. ramosa-*exposed replicates was 599 ± 80 (*n* = 24), whereas control replicates had a mean of 196 ± 11 circulating haemocytes (*n* = 24). However, the magnitude of the parasite-induced cellular response depended on the identity of the host genotype: i.e. there was a parasite exposure by host genotype interaction (figure 2 and table 1). When genotype is coded as a random effect, parasite exposure remains significant (*F*_1,3_ = 15.26, *p* < 0.05), and a model with the parasite exposure-by-genotype effect explained significantly more variation than did a model without the interaction term (*χ*^2^ = 4.60, d.f. = 1, *p* < 0.05).

**Table 1.** Summary of analysis of the number of circulating haemocytes in an experiment involving four host genotypes of *D. magna*. The effects tested were parasite (exposed or not), time post-exposure and host genotype.

number of haemocytes	d.f.	*F*	*p*	% var ^a^
time	3	2.18	0.09	2.19
parasite	1	61.31	<0.0001	20.57
genotype	3	11.13	<0.0001	11.2
time × parasite	3	1.82	0.14	1.84
time × genotype	9	1.09	0.37	3.29
parasite × genotype	3	4.02	<0.01	4.05
time × parasite × genotype	9	1.05	0.40	3.18
error	160			53.69

^a^Percentage of the total variance explained by each term in the full model.

### (b) 16-genotype cell experiment

**Figure 3.**
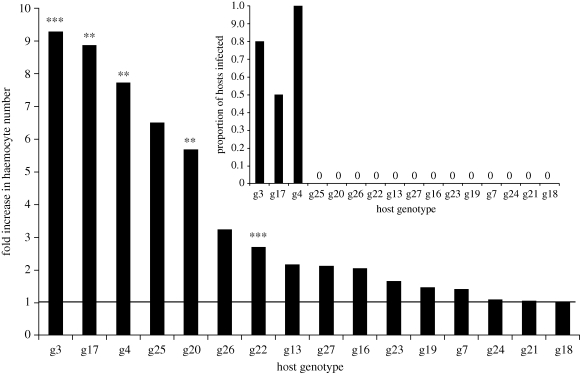
Fold induction of haemocyte numbers in *P. ramosa-*exposed *D. magna* (*n* = 6, each replicate consists of five *Daphnia*), relative to unexposed *D. magna* (*n* = 6, each replicate consists of five *Daphnia*). The bold line at *y* = 1 shows the uninduced (basal) level. The inset shows the proportion of individuals that became infected in *P. ramosa*-exposed treatments in each genotype (*n* = 12, each replicate consists of an individual *Daphnia*). Asterisks indicate if haemocyte numbers rose significantly (after sequential Bonferroni adjustment) above basal levels: ** *p* < 0.01, *** *p* < 0.001.

Haemocyte counts were obtained from 960 *Daphnia* from 192 jars. As before, a cellular response followed *P. ramosa* exposure, with a mean per microlitre haemocyte count that was highly consistent with the previous experiment: 614 ± 50 cells for *P. ramosa-*exposed replicates (*n* = 96) and 208 ± 17 haemocytes per microlitre for control jars (*n* = 96). Basal haemocyte counts differed across host genotypes (*F*_15,80_ = 4.49, *p* < 0.001); and, there was also considerable genetic variation in the magnitude of cellular response, varying between a one and ninefold increase in haemocyte number depending on the identity of the host genotype (figure 3). Statistically, this appears as a strong parasite exposure by host genotype interaction (table 2). The three host genotypes that mounted the strongest cellular response were the three genotypes that suffered infection from *P. ramosa* (figure 3). Again, the parasite treatment remains significant with genotype as a random effect (*F*_1,15_ = 27.76, *p* < 0.0001), and the parasite exposure-by-genotype effect explained significantly more variation than did a model without the interaction term (*χ*^2^ = 32.86, d.f. = 1, *p* < 0.0001).

**Table 2.** Summary of analysis of the number of circulating haemocytes in an experiment involving 16 host genotypes of *D. magna*. The effects tested were parasite (exposed or not) and host genotype.

number of haemocytes	d.f.	*F*	*p*	% var^a^
parasite	1	157.29	<0.0001	28.53
genotype	15	9.72	<0.0001	26.67
parasite × genotype	15	5.67	<0.0001	15.54
error	160			29.26

^a^Percentage of the total variance explained by each term in the full model.

*Post hoc* tests revealed a significant cellular response, i.e. that the number of circulating haemocytes was greater in exposed versus unexposed in the following five host genotypes: 3, 4, 17, 20 and 22 (figure 3). This was after the data were corrected using the sequential Bonferroni adjustment (Holm 1979). Of these five responding genotypes, three suffered infection from *P. ramosa* (3, 4 and 17).

### (c) 16-genotype life-history experiment

Successful infection was recorded in three of the 16 genotypes, where infection with *P. ramosa* caused a substantial reduction in the number of offspring produced by the *Daphnia*. Of replicates from the parasite-exposed treatment, uninfected hosts had 48.05 ± 0.78 offspring, whereas infected hosts had 32.21 ± 1.15 offspring (*t* = 11.35, d.f. = 47.61, *p* < 0.0001).

### (d) Killed parasite cell experiment

Haemocyte counts were obtained from 120 *Daphnia* from 24 jars. The strongest cellular response followed exposure to live parasite spores, with a mean haemocyte count of 584 ± 83 haemocytes per microlitre for live *P. ramosa-*exposed jars (*n* = 8) and 65 ± 13 for control jars (*n* = 8). There was also a smaller but significant cellular response from jars exposed to heat-treated *P. ramosa* spores: 238 ± 30 haemocytes (*n* = 8). *Post hoc* tests revealed that haemocyte counts from all treatments were significantly different from each other (Tukey's HSD, *p* < 0.05). Only jars exposed to live *P. ramosa* spores went on to develop infection (data not shown).

## 4. Discussion

Just hours after exposure to the bacterial parasite *P. ramosa*, there was a large increase in the number of amoeboid cells circulating in the haemolymph of *D. magna*. These data also revealed very large differences in cellular response between host genotypes, ranging from no increase to a greater-than ninefold increase in cell number (figures 2 and 3). Basal (uninduced) haemocyte counts did differ across host genotypes, but these differences did not predict the likelihood of becoming infected. This differs from the finding that *Drosophila melanogaster* with a greater basal haemocyte level were more resistant to parasitoid infection (Kraaijeveld *et al*. 2001). Non-infective parasite spores (i.e. those we heat-killed prior to exposure) elicited a small increase in the number of circulating haemocytes, suggesting that the presence of parasite material in the gut may trigger weak immune reactions; perhaps bacterial ligands are penetrating the gut mucosa and triggering an immune response (Raz 2010). However, data from the killed-spore experiment clearly show that live infective spores induce a much stronger cellular response.

This cellular response is possibly the host immune response that the parasite encounters when it passes from the host gut into its body, supporting very early work showing *D. magna* mounts a cellular response to a yeast-like infection (Metchnikoff 1884). Immune function and immunity, however, are clearly *not* one and the same: the largest increase in cell numbers was seen in the host genotypes that were susceptible to the parasite (figure 3). Other studies of putative immune responses found no link between infection status and strength of the response (e.g. Mucklow *et al*. 2004). If the cellular immune response to *P. ramosa* depends upon the parasite spores passing the gut epithelium, complete resistance appears to be achieved by preventing that passage (as opposed to destroying parasites once they have gained access). A very strong cellular response thus appears to be indicative of a critical failure elsewhere in the host immune system (most likely in the gut epithelium), and it appears that the gut epithelium forms the main defence.

The *P. ramosa* infection process may be similar to that seen in *Pasteuria penetrans*, a sterilizing parasite that initiates infections by attaching to the heparin-binding domain and gelatine-binding domain proteins on the cuticle of *Meloidogyne* nematodes (Sayre & Starr 1985; Mohan *et al*. 2001; Schmidt *et al*. 2008). The external surface of the nematode encounters *P. penetrans* as it migrates through the soil, whereas *P. ramosa* is thought to be taken up as the *D. magna* filter feed where it then penetrates the gut. Aside from this difference in the location of infection, *P. ramosa* may similarly require binding to epithelial proteins to initiate infection, and without this binding the infection process, subsequent cellular response will not occur. The probability of molecular binding to *D. magna* epithelial proteins appears to be subject to host genetic variation; or, there is variation in other gut-based defences. We propose that a lack of molecular matching explains cases of resistance, while a strong cellular response indicates a molecular genetic match that allows parasites to overcome gut defences. This heuristic model of a two-tiered defence is largely supported by the observation that the three susceptible host genotypes had the strongest cellular responses, while the majority of non-responding genotypes remained healthy (figure 3). Still, two host genotypes responded to parasite exposure but showed no signs of infection, which indicates that the cellular immune response may only play a limited role in resistance, if only a very small number of spores reach the haemolymph.

Previous work has modelled the genetics of infection as a two-stage process, with ‘matching-allele’ genetics for parasite detection, and ‘gene-for-gene’ genetics for parasite eradication (Agrawal & Lively 2003). *Daphnia magna*'s patterns of resistance and cellular responses to *P. ramosa* can be used to test such models. Thus, a desirable follow-up study to the present work comparing host genotypes would be experiments incorporating both host and parasite genetic variation (*sensu* Carius *et al*. 2001), as well as with parasites from other taxa, where a cellular response may successfully provide resistance. Studies of such genetic specificity and the cellular response would be the next step towards elucidating the immunological basis of invertebrate coevolutionary interactions.

A substantial body of work in invertebrate immunology has studied the response to opportunistic bacteria, generalist entomopathogens or chemical pathogen mimics (e.g. LPS); and there are considerable merits in measuring immune function in non-coevolved systems (Barnes & Siva-Jothy 2000), primarily that the parasite has not had the opportunity to evolve avoidance of host immune responses (Huxham *et al*. 1988; Barnes & Siva-Jothy 2000). By adopting such an approach one can better assess the generality of a host's immune function without the confounding influence of anti-parasite defence mechanisms. Conversely, our use of a naturally coevolving host–parasite combination means the cellular response we document reflects how invertebrates defend themselves against natural enemies. Indeed, outside of the well-studied interaction between mosquitoes and *Plasmodium* parasites, we have little understanding of the invertebrate immune response to coevolving biological enemies. Thus, in the study of invertebrate immunity, our work is a rare example of the (putative) immune response and genetic variation for that response, against a natural parasite.

It is now widely acknowledged that a stronger immune response does not necessarily lead to higher fitness—the relationship between host fitness and both size of immune response and parasite burden may not be linear (Adamo 2004; Viney *et al*. 2005; Stjernman *et al*. 2008). Our work is a compelling example of this point: had we measured only haemocyte responsiveness without assessing infection probabilities (and hence fitness), a misleading impression of which is the fittest genotype would have emerged. This argues against the practice (common in the early days of ecological immunology) of measuring immune parameters in isolation from infection biology. Moreover, the large differences in cellular response between host genotypes emphasizes the need to embrace genetic variation when studying immune function. Had we looked for a cellular response in just one host genotype, our results would very much depend on which genotype we studied. For example, a study of host genotype 3 would lead to opposite conclusions to a study of genotype 18. This makes clear the need to effectively link studies of immune function to studies of infection outcome in multiple host genotypes. That being so, the next stage is to investigate the role of parasite genetic variation: both how it modifies cellular response in different host genotypes, and how this links to infection outcome.
